# Special problems encountering surgical management of large retroperitoneal schwannomas

**DOI:** 10.1186/1477-7819-6-107

**Published:** 2008-10-03

**Authors:** Theodosios Theodosopoulos, Vaia K Stafyla, Paraskevi Tsiantoula, Anneza Yiallourou, Athanasios Marinis, Agathi Kondi-Pafitis, Achilleas Chatziioannou, Efstathios Boviatsis, Dionysios Voros

**Affiliations:** 1 Second Department of Surgery, Areteion Hospital, University of Athens, Greece; 2 Department of Pathology, Areteion Hospital, University of Athens, Greece; 3 Department of Radiology, Areteion Hospital, University of Athens, Greece; 4 Department of Neurosurgery, "Evangelismos" General Hospital, Athens, Greece

## Abstract

**Background:**

Retroperitoneal schwannomas are rare, usually benign tumors that originate in the neural sheath and account for only a small percentage of retroperitoneal tumors. The aim of this clinical study is to present our experience in managing retroperitoneal schwannomas with a review of the current literature and to point out the surgical technical difficulties we faced, due to the tumor's strange behavior that eroded the vertebra in two cases without causing malignant invasion.

**Methods:**

We reviewed the medical files of 69 patients treated in our department for retroperitoneal tumors from January 1991 until December 2006. Five patients had retroperitoneal schwannomas according to pathology report.

**Results:**

There were two male and three female patients, with a mean age of 56 years (range 44–67 years). All patients were asymptomatic and none suffered from von Recklinghausen disease. Imaging workup included ultrasonography, computed tomography and magnetic resonance imaging. One patient, after having a non-diagnostic computed tomography fine needle aspiration (CT-FNA), underwent exploratory laparotomy and incisional biopsy that established the diagnosis of schwannoma. After complete excision of the tumors, postoperative course was uneventful in all patients. Tumors' maximum diameter was 12.7 cm (range 7–20 cm). No recurrences were detected during the follow up period (6–75 months).

**Conclusion:**

Preoperative establishment of diagnosis is difficult in case of retroperitoneal schwannomas, however close relationship of retroperitoneal tumors with adjacent neural structures in imaging studies should raise a suspicion. Complete surgical resection is the treatment of choice. Histology and Immunohistochemistry confirms the diagnosis.

## Background

Neural sheath tumors are a subclass of soft tissue neoplasms that include both benign and malignant schwannomas and neurofibromas. Schwannomas are found most commonly in cranial and peripheral nerves and occur rarely in the retroperitoneum, the last comprising about 3% of all schwannomas [1]. Schwannomas constitute approximately 4% of all retroperitoneal tumors [2-4]. They are typically solitary, circumscribed and encapsulated lesions on gross appearance [5]. Histologically, schwannomas are distinguished by the presence of areas of high and low cellularity, called Antoni A and B tissue, respectively [6]. They are often found incidentally, or present with vague, non specific symptoms.

In this study, clinical, imaging and histological characteristics, but mainly the treatment of five retroperitoneal schwannomas, are analyzed with a review of the literature.

## Methods

Sixty nine (69) patients with retroperitoneal tumors were treated in our department between January 1991 and December 2006. Five of them had retroperitoneal schwannomas. Preoperative imaging workup included abdominal ultrasound (U/S), computed tomography (CT) and magnetic resonance imaging (MRI). Pheochromocytomas were excluded by specific studies (urine catecholamines and MIBG). Treatment in all cases was complete resection of the mass, as well as en bloc excision of any involved adjacent structures or organs, when necessary. The diagnosis of schwannoma was based on detection of Schwann cells with Antoni A and B regions in histological sections and positive staining for S-100 protein in immunohistochemical analysis. Review of the literature was based upon research in PubMed.

## Results

### Case 1

**Figure 1:**
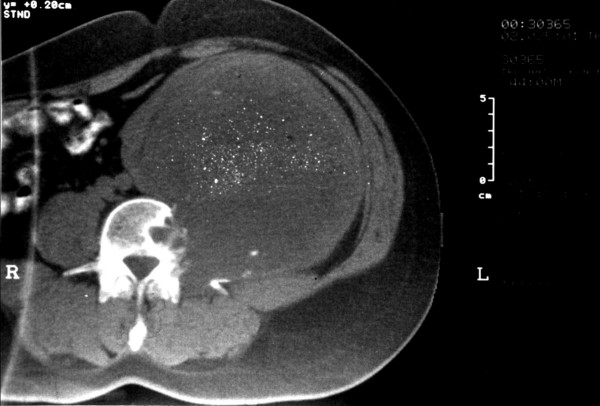
CT scan shows a 13.5 × 12 cm retroperitoneal mass eroding the left side of the 4^th ^lumbar vertebrae.

A 44-year-old male patient presented to us with a palpable mass, measuring 13,5 × 12 cm by CT, which was extending from the left upper quadrant to the left iliac crest, with co-existing erosion of the left side of the 4^th ^lumbar vertebra (Fig. 1) whereas the bone scan was negative. MRI showed the mass to protrude from the 4^th ^lumbar vertebral foramen, indicating its possible origin from the corresponding nerve, with no evidence of intraspinal extension. We excised the mass en bloc with part of the left psoas muscle. Small amount of residual tumor, approximately 1 cm, was left along the root of the 4^th ^lumbar nerve. The patient recovered uneventfully from the operation and was referred to neurosurgeons for the residual tumor. They decided only to follow him up and he remains disease free for 75 months without any enlargement of the residual tumor or any significant corresponding symptomatology.

### Case 2

A 67-year-old female patient complained of vague abdominal discomfort and vaginal hemorrhea. U/S revealed a pelvic mass with mixed echogenicity measuring 7.6 × 6.9 cm that was also confirmed by CT scan. The mass was attached to the posterior wall of the uterus and the patient underwent laparotomy with total abdominal hysterectomy and en bloc tumor excision. Interestingly, pathology revealed the uterus with invasion of a low differentiated endometrial adenocarcinoma, for which she received adjuvant chemotherapy. The patient had an uneventful postoperative course and is disease free for 48 months.

### Case 3

**Figure 2:**
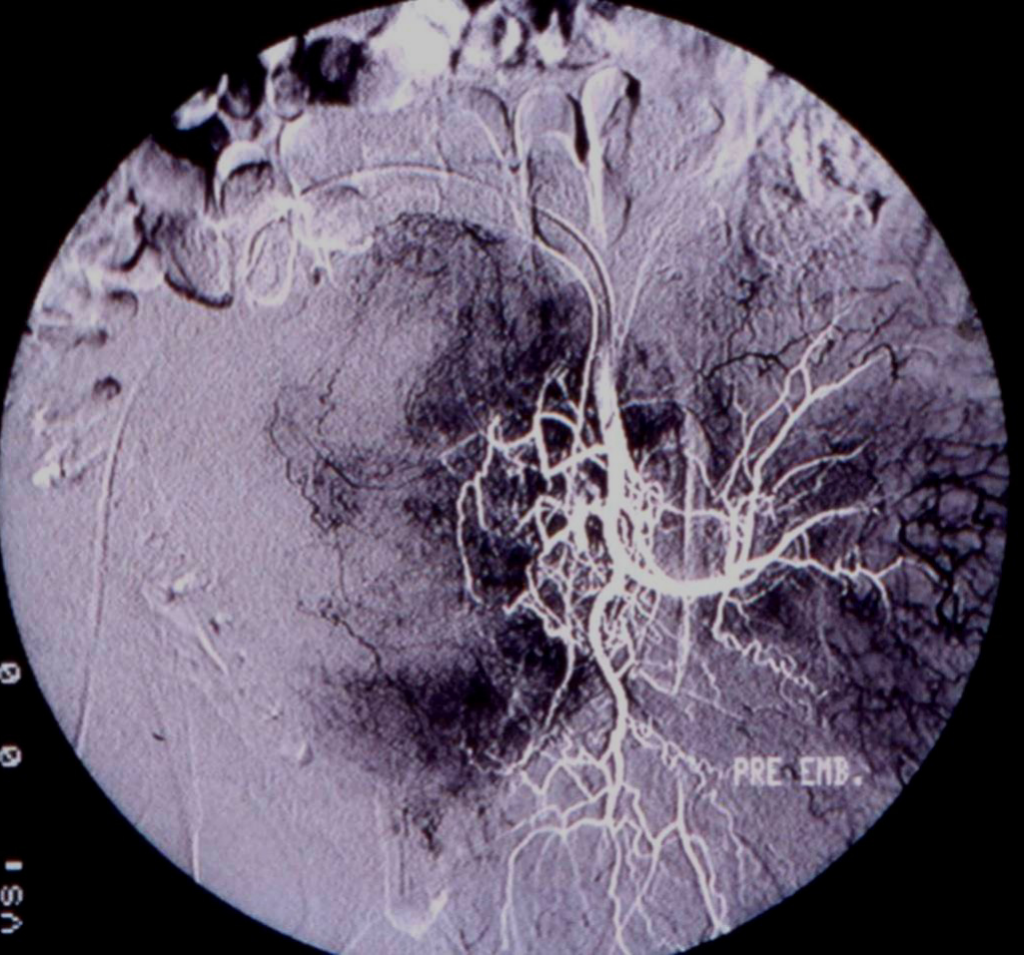
Pre-embolism angiography shows the tumor's vascularity.

A 53-year-old male patient presented with deep venous thrombosis of the left leg. U/S revealed a solid, well circumscribed mass in the left retroperitoneal space with mixed echogenicity, trapping the left iliac vessels and the left ureter. CT scan showed a 19.5 × 13.6 × 12.6 cm heterogeneous mass located in the presacral space displacing the left iliac vessels, the sigmoid colon and the left ureter towards the midline. Significant thrombosis of the left iliac and femoral veins was identified. The CT guided FNA biopsy that followed was non diagnostic. We didn't perform core-needle biopsy, because the patient was under anticoagulation for the vein thrombosis. The findings of the MRI were similar to the CT. Due to extended deep vein thrombosis a filter was placed in the inferior vena cava before any surgical management. An exploratory laparotomy followed and the large retroperitoneal tumor was found adherent to the sacrum and displacing the urinary bladder and the rectosigmoid colon. After two and a half hours effort to separate the tumor from the viscera, it was considered unresectable because of dense attachment to the sacrum. An incisional biopsy was obtained at this stage. A complete embolization of the tumor from both internal iliac arteries, in order to reduce its size, was performed with PVA particles (250–355 microns) (Fig. 2). Ten days after the initial laparotomy and 6 days post embolization a reexploration was carried out and even though the size was more or less the same, the tumor was quite soft, mobilized easier from the sacrum and removed totally. The patient's postoperative course was uneventful. During the 37 month follow up period the patient is disease free without any symptoms of deep venous thrombosis.

### Case 4

A 52-year-old female patient complained of right flank discomfort and constipation. U/S revealed a well encapsulated, circumscribed mass located in the retroperitoneum. The mass had mixed echogenicity and measured 7.8 × 6.2 cm. CT scan showed a heterogeneous retroperitoneal mass measuring 8 × 6 cm, that was adherent to the right psoas muscle. MRI showed an 8 cm retroperitoneal tumor with hypointensity on T1 and heterogeneous hyperintensity on T2 weighted images. The solid peripheral elements of tumor were enhanced after intravenous gadolinium administration. During laparotomy a retroperitoneal tumor was found, located behind the ascending colon and adherent to the right psoas muscle. We performed a complete excision of the tumor with part of the psoas muscle and part of the adherent nerve. Postoperatively, the patient reported hypoesthesia on the medial surface of the right leg and weakness of the distal muscle. During a 9-month follow up period, the patient is disease free.

### Case 5

A 63-year-old female patient presented with left flank pain. MR imaging revealed a retroperitoneal mass measuring 8.5 × 5.8 cm with erosion of the left side of the 4^th ^lumbar vertebrae whereas the bone scan was negative for vertebral invasion. The mass was homogenously hypointense on T1 and heterogeneously hyperintense on T2 weighted images with intense enhancement after gadolinium administration. With the CT angiography that followed we clarified the tumor's blood supply that was originating from the superior lumbar artery. During the laparotomy we found a retroperitoneal mass that had eroded the left side of the 4^th ^lumbar vertebra and displaced the ipsilateral ureter and psoas muscle. The tumor was completely excised, while the vertebra was left intact with erosion of its left side due to tumor's pressure. The bone cavity that remained in the vertebral body was filled with bone wax. The patient had an uncomplicated recovery and remains free of recurrence during the 6-month follow up.

## Discussion

Schwannomas or neurilemomas are neoplasms that arise from Schwann cells of nerve sheaths and belong to the category of neural sheath tumors. They can be found in any nerve trunk, except for cranial nerves I and II, and their usual location is the head, neck, the flexor surfaces of the extremities and the posterior mediastinum or the retroperitoneum [7,8]. There is a controversy in the literature about the gender predominance. In one large series with 895 cases by Kransdorf, men predominate [9] and in contrast with other retroperitoneal tumors they appear single without any satellite lumps [10].

Schwannomas are usually benign and are associated with von Recklinghausen disease in 5–18% of cases [11]. Malignancy is very rare and is usually observed in patients with von Recklinghausen disease [12,13]. In our series none of the patients had von Recklinghausen's disease and all schwannomas were benign. Retroperitoneal schwannomas comprise 3% of all schwannomas according to the literature and present in patients in their third and fourth decades of life [1,4]. Of all benign schwannomas only 0.3–3.2% are retroperitoneal [14]. These neoplasms are usually large, 10–20 cm in diameter, by the time of surgery, because they are mostly asymptomatic and patients report non-specific symptoms, such as vague abdominal or back pain and discomfort, something that is true for our series, too [[5,15] and [16]]. Atypical presentations of retroperitoneal schwannomas, such as headache and secondary hypertension or renal colic pain with hematouria have been reported [17,18]. Benign schwannomas do not invade adjacent organs, so the symptoms are due to organ "displacement" in the retroperitoneal space and concern mainly the GI tract and the urinary system. Our patients were symptomatic and the tumors were displacing the rectosigmoid, the ureter, the uterus and the psoas muscle. Interestingly, in two cases the schwannoma actually eroded a lumbar vertebra, but did not infiltrate it. Review of the literature revealed sporadic cases of vertebral involvement, with a high incidence of L5 nerve root encasement due to the long length and large size of them [19].

Preoperative diagnosis based on clinical examination is very difficult and so the role of imaging is important. Ultrasonography is a cheap modality for revealing a mass with semisolid or cystic areas, but it is not used widely due to specificity limitations [20,21]. Mixed echogenicity was a common feature in our cases. CT scan and MRI are more helpful in detecting specific characteristics of the tumor. Size, exact location, relationship with other organs and invasion can be accurately reproduced [22,23]. Calcifications and tumor heterogenicity due to cystic degeneration – that reaches up to 66% – may also be seen and characterizes a special type called "ancient schwannoma" [24,25]. MRI is the examination of choice and presents iso- or slightly hyper-intensity on T1 weighted images according to the literature. In contrast, in our cases the tumors presented hypointense on T1 and hyperintense on T2 weighted images. Compared to CT scan, MRI has higher specificity, better resolution and can delineate the tumor better, but still it can not distinguish between benign and malignant tumors [26,27]. Angiography has also been reported by some authors because of the hypervascularity of these tumors and the possibility of embolization, but is not widely used. In one of our cases we performed preoperative angiography and arterial embolization in an attempt to reduce the size of the mass and in another case an angiography, in order to obtain details about tumor blood supply.

Despite these accurate imaging techniques, the definite diagnosis of retroperitoneal schwannoma is uncertain and the surgeon should include in the preoperative differential diagnosis other tumors, such as neurofibroma, paraganglioma, pheochromocytoma, liposarcoma, malignant fibrous histiocytoma, and hematoma. CT-guided biopsy is a possible modality that can establish a preoperative diagnosis, under the limitation that the sample contains enough Schwann cells and not degenerative cells obtained from areas of cellular pleomorphism that can be misleading. CT-guided FNA is usually unsuccessful and unreliable. CT-guided core needle biopsy seems to have better results despite the existing controversy in literature. Some authors suggest that this diagnostic modality may not only be inconclusive, but may also have a high risk of tumor seeding, hemorrhage, and infection. For these reasons they encourage incisional biopsy, while others report interesting results in establishing a preoperative diagnosis [28]. We performed a CT guided FNA biopsy in one case, but it was non diagnostic.

The pathologic examination of the tumor specimen reveals microscopically elongated bipolar spindle cells with a focal nuclear palisading pattern. There are areas of high cellularity named Antoni A and with low cellularity and myxoid matrix named Antoni B. This finding is suggestive of the benign nature of the tumor. Immunohistochemistry shows NSE, microfilament proteins and S-100 protein, which is the neural protein within the Schwann cell that differentiates schwannomas from neurofibromas, since the latter do not express it due to their perineural origin [29]. Histological and Immunohistochemical studies in our patients showed a mean maximum diameter of the schwannomas of 12,7 cm (7–20 cm), with areas of degeneration (cases 2, 4 and 5) and atypia (cases 1 and 3), while all were positive for vimentin and S-100 and negative for desmin, smooth muscle actin and HHF35.

A variant of the typical schwannoma is the "ancient type" or "degenerative neurilemoma" that presents with features of degeneration, cystic changes and hyalinization [[24,25] and [29]]. In some of these tumors nuclear atypia and hyperchromatism may be suggestive of malignant transformation, although it is extremely rare. In the case of malignancy, nerve sheath neoplasms act as high grade sarcomas and are characterized histologically by dense fascicles in a "marble-like" pattern consisting of asymmetrically tapered spindle cells.

The surgical approach to retroperitoneal schwannomas remains debatable. It is well known that local recurrence and malignant transformation of retroperitoneal schwannomas in absence of von Recklinghausen disease is extremely rare, so local excision should be the treatment of choice, sparing the adjacent vital organs [7,13]. In this setting, some authors performed simple enucleation of the tumor with good results [30]. Others believe that since malignancy can not be excluded preoperatively, or with intra-operative frozen section, the surgeon should obtain clear margins even if other organs have to be sacrificed. It is true that, in case of malignancy after marginal excision local recurrence is 72%, versus 11.7% after wide margin resection [14,31].

There are also some reports of laparoscopic resections [32,33]. Hemorrhage is a serious intraoperative problem in cases that major vessels are situated nearby the tumor and there are several reports of unsuccessful tumor excision or even intra-operative death.

In our series we followed the approach of the radical resection of the tumor instead of enucleation assuming that we had to deal with a retroperitoneal tumor of unknown pathology. Based on this hypothesis, in order to ensure the optimum treatment and survival for our patients we performed laparotomy and complete excision with wide margins [34]. In spite of the vicinity of tumors to vital retroperitoneal structures, such as the aorta, the inferior vena cava, the renal and iliac vessels and peripheral nerves, careful dissection and manipulation of them was carried out. An en bloc resection of the schwannoma and adjacent organs was performed in three cases, two with psoas muscle and one with the uterus. In our series all the patients are disease free during follow up. Of course, these problems remain to be studied and evaluated in a larger number of cases.

## Conclusion

We think that in our cases there are two points of interest. Firstly, the erosions of the lumbar vertebrae were due to tumor pressure and not invasion. Secondly the beneficial effect of tumor embolization in one case which according to our knowledge from the literature is not a widely or routinely practice.

## Competing interests

The authors declare that they have no competing interests.

## Authors' contributions

TT was responsible for critical revision of scientific content. VKS drafted the manuscript. PT participated in the design of the manuscript and helped to draft the manuscript. AY contributed substantially to manuscript conception and design. AM assisted in the preparation of the manuscript. APK performed histopathological and immunohistochemical analyses and contributed to the pathology content. AC performed the embolization of one of the tumors, the filter placement and have made substantial contributions to manuscript conception and design.EB participated in one of the surgical operation and participated in the acquisition of data and preparation of the manuscript. DV the surgeon, approved the final version of the manuscript for publication.
